# Turgor-responsive starch phosphorylation in *Oryza sativa* stems: A primary event of starch degradation associated with grain-filling ability

**DOI:** 10.1371/journal.pone.0181272

**Published:** 2017-07-20

**Authors:** Hiroshi Wada, Chisato Masumoto-Kubo, Koichi Tsutsumi, Hiroshi Nonami, Fukuyo Tanaka, Haruka Okada, Rosa Erra-Balsells, Kenzo Hiraoka, Taiken Nakashima, Makoto Hakata, Satoshi Morita

**Affiliations:** 1 Kyushu Okinawa Agricultural Research Center, National Agriculture and Food Research Organization, Chikugo, Fukuoka, Japan; 2 Department of Biomechanical Systems, Faculty of Agriculture, Ehime University, Matsuyama, Ehime, Japan; 3 Central Region Agricultural Research Center, National Agriculture and Food Research Organization, Tsukuba, Ibaraki, Japan; 4 Department of Organic Chemistry-CIHIDECAR, Faculty of Exact and Natural Sciences, University of Buenos Aires, Buenos Aires, Argentina; 5 Clean Energy Research Center, The University of Yamanashi, Kofu, Yamanashi, Japan; Kyung Hee Univeristy, REPUBLIC OF KOREA

## Abstract

Grain filling ability is mainly affected by the translocation of carbohydrates generated from temporarily stored stem starch in most field crops including rice (*Oryza sativa* L.). The partitioning of non-structural stem carbohydrates has been recognized as an important trait for raising the yield ceiling, yet we still do not fully understand how carbohydrate partitioning occurs in the stems. In this study, two rice subspecies that exhibit different patterns of non-structural stem carbohydrates partitioning, a *japonica*-dominant cultivar, Momiroman, and an *indica*-dominant cultivar, Hokuriku 193, were used as the model system to study the relationship between turgor pressure and metabolic regulation of non-structural stem carbohydrates, by combining the water status measurement with gene expression analysis and a dynamic prefixed ^13^C tracer analysis using a mass spectrometer. Here, we report a clear varietal difference in turgor-associated starch phosphorylation occurred at the initiation of non-structural carbohydrate partitioning. The data indicated that starch degradation in Hokuriku 193 stems occurred at full-heading, 5 days earlier than in Momiroman, contributing to greater sink filling. Gene expression analysis revealed that expression pattern of the gene encoding α-glucan, water dikinase (*GWD1*) was similar between two varieties, and the maximum expression level in Hokuriku 193, reached at full heading (4 DAH), was greater than in Momiroman, leading to an earlier increase in a series of amylase-related gene expression in Hokuriku 193. In both varieties, peaks in turgor pressure preceded the increases in *GWD1* expression, and changes in *GWD1* expression was correlated with turgor pressure. Additionally, a threshold is likely to exist for *GWD1* expression to facilitate starch degradation. Taken together, these results raise the possibility that turgor-associated starch phosphorylation in cells is responsible for the metabolism that leads to starch degradation. Because the two cultivars exhibited remarkable varietal differences in the pattern of non-structural carbohydrate partitioning, our findings propose that the observed difference in grain-filling ability originated from turgor-associated regulation of starch phosphorylation in stem parenchyma cells. Further understanding of the molecular mechanism of turgor-regulation may provide a new selection criterion for breaking the yield barriers in crop production.

## Introduction

In plant cells, turgor pressure plays a central role in most physiological processes. The regulation of turgor pressure is, therefore, the key to understanding cellular metabolism during development and responses to changes in environmental conditions. Some studies using the green algae *Chara corallina* have addressed the role of turgor pressure in cell enlargement [1–3]. It has been also reported that in grape (*Vitis vinifera*) fruits decreases in turgor pressure and softening were involved in the regulation of both sugar metabolism and anthocyanin biosynthesis at the onset of ripening [4]. Even with several lines of evidence, how exactly turgor pressure is physically involved in transcriptional and metabolic changes remains questionable. Extensive research on metabolic regulation has been conducted at the molecular level, although in most cases, accurate water status measurements have been lacking.

One reason for this might be the technical limitations of instruments for measuring the water status of plants. For example, thermocouple psychrometers that employ a vapour pressure equilibration method for measuring tissue water potential, as well as the pressure chambers, have been widely used. Of these, the isopiestic psychrometers are the most reliable and allow the determination of the water potential, osmotic potential, and turgor pressure of the same sample [5], but at present they are commercially unavailable. Unfortunately, the other psychrometers have not been improved and are less accurate than the isopiestic psychrometers. Another reason is that the number of researchers who specialize in plant-water relations and are capable of operating the isopiestic psychrometers has decreased over the past three decades. However, in addition to molecular approaches, the water status measurements appeared to be important, as indicated by Boyer [6]. Such combined analyses are necessary to investigate turgor regulation during physiological processes, such as carbohydrate partitioning in field crops.

Crop production is required to increase by >70% by 2050 to match the human population growth worldwide [7]. Rice (*Oryza sativa* L.) is a staple crop that supplies energy to over half of world’s population, and hence, establishing high-yielding varieties (HYVs) with greater yield stability is an urgent requirement in rice production under climate change [8]. To promote breeding programs, the development of robust high-throughput screening method(s) has become important for rice breeding. Various attempts, such as improving nitrogen use efficiency, light interception, and water use efficiency, have been made to increase yield potential. In addition, development of functional genomic resources for yield traits [9], including altering the photosynthetic pathway of rice [10], has been studied. QTL/genes of transport efficiency are another potential trait that has been desired to incorporate into breeding [11]. It is generally known that approximately 30% of grain carbohydrates are derived from non-structural carbohydrates (NSC) in stems (leaf sheaths and culms) under normal conditions [12]. Grain-filling ability is determined by the availability of stem NSC during the initial 10 days of grain filling, corresponding to the period in which the number of endosperm cells is determined [13]. NSC accumulated in stems at full-heading has long been recognized as an important high-yield trait for breeding [14]. Nevertheless, understanding the underlying mechanism(s) of NSC partitioning from stems to kernels has remained obscure. Hence, reliable selection criteria regarding NSC partitioning have not been established in breeding programs [11].

Cultivars with relatively large spikelets, such as New Plant Type (NPT) rice [15], ‘super’ rice [16], and *japonica*-dominant HYVs (e.g., Akenohoshi [17, 18]) tend to exhibit poor grain-filling because of lower grain filling in inferior kernels. A clear subspecies difference in seasonal patterns of stem NSC accumulation between two major high-yielding subspecies (*indica-* and *japonica*-dominant rice) has been reported [17]. The rapid translocation of stem NSC consistently observed in *indica-*dominant HYVs was attributed to higher grain-filling ability compared with *japonica*-dominant HYVs [17]. A recent paper reported a similar trend in starch content in leaf sheaths in a *japonica*-dominant variety, Nipponbare, and an *indica*-dominant HYV, Takanari, and concluded that an increase in amylase activity in Takanari played a role in rapid starch degradation [18]. Considering the recent advances on starch phosphorylation in starch metabolism [19, 20], it is possible that prior to the amylase activity starch phosphorylation might occur at the surface of starch granules in amyloplasts, although to the best of our knowledge, the regulatory mechanism of starch phosphorylation is still unknown in rice plants.

For transcriptional regulation of nuclear gene expression, it is generally recognized that RNA polymerase II binds to the promoter region and requires general transcription factors and other regulatory proteins to initiate gene expression [21]. There is a large body of literature describing that water molecules physico-chemically influence the structure, stability, dynamics, and function of biological macromolecules, such as protein-DNA and protein-protein complexes [22–24]. It has been reported that water mediates recognition and specificity as a hydrogen donor and acceptor at interface of protein-DNA complexes, and the binding process is accompanied by the release of water molecules and counterions as well as changes in DNA-protein conformations [24]. In the cells, the volume of both nuclei and chloroplasts are shown to be osmotically sensitive [25, 26]. Furthermore, it has been reported that 0.2 MPa of pressure differences induce plasmodesmatal closure in *Nicotiana clevelandii* cells [27]. Significant decreases in membrane hydraulic conductivity have been observed at 1 min after 0.1–0.2 MPa of hydraulic disturbance was instantly given [28, 29]. Hence, if turgor pressure was altered in a cell, the hydraulic pressure differential would be rapidly transmitted to the nucleus and other organelle compartments. In this view, we have hypothesized that turgor pressure affects nuclear gene expression during the volume change.

In this study, two *indica*- and *japonica*-dominant HYVs, Hokuriku 193 (H193) and Momiroman (MOMI) were used to study the possible interaction between turgor pressure and the transcriptional regulation of stem NSC partitioning. We combined plant water status measurements with a genomic DNA-based absolute quantification [30] of target genes in the stems, including starch synthesis and degradation-related genes, to exclude possible variation in expression of the reference gene(s) that potentially exists under fluctuating environmental conditions during the early ripening stage. Furthermore, a pre-fixed ^13^C tracer analysis at the whole-plant level [31] was carried out to identify subspecies differences in the timings of starch degradation by tracing ^13^C-labeled Glc, once spatially synthesized in stem starches at pre-heading, at the certain time(s) during the post-heading stage. Herein, we show that there may be a subspecies difference for turgor-responsive starch phosphorylation at the initial step of stem NSC partitioning. We also report that turgor-associated starch phosphorylation that initially occurs in stem storage parenchyma cells would be responsible for the onset of stem NSC partitioning that determines grain-filling ability between *indica*- and *japonica*-dominant HYVs [17].

## Results

### Yield and yield components

When the heading dates of two varieties were synchronized in the field, the average rough grain yield of H193 was overall 8.6% greater than that of MOMI; a similar finding occurred for hulled rice (Table 1). Yield level was relatively low in 2013 presumably because of higher temperatures at the stage between panicle initiation and full heading compared to that of the other two years (S1 Table). Mean panicle number for MOMI was 11% lower than that for H193, but the number of spikelets per panicle in MOMI tended to be larger than that in H193. Consequently, the number of spikelets in MOMI was similar to that in H193 with no interaction between cultivar and year (Table 1). The percentage of ripened grains in MOMI was consistently lower than that in H193 with no interaction between cultivar and year (Table 1). 1000-grain weight in H193 was smaller than that in MOMI. Also, the panicle weight increase in H193 was prone to be higher than MOMI at the ripening stage (S1 Table), suggesting that H193 exhibited greater grain growth rate than MOMI during the early ripening stage. Furthermore, sink capacity in MOMI was greater than that in H193 even with year-to-year variations (Table 1). Conversely, sink filling in MOMI was consistently much lower (between 76 and 86%) than that in H193 (Table 1). Within-panicle analysis showed that the observed lower sink filling was caused by poor filling rate on the tertiary pedicels, regardless of the year effect (S2 Table).

**Table 1:** Yield and yield components of Hokuriku193 (H193) and Momiroman (MOMI) in 2012–2014. Sink capacity was calculated as single grain weight multiplied by the number of spikelets per area. Sink-filling rate was calculated as the hulled grain yield divided by sink capacity. Values for ripened grains and sink filling were transformed by using Box-Cox procedure prior to the analysis. ns indicates not significant by ANOVA.

Year	Cultivar	Sowing date	Transplanting date	Heading date	Rough grain yield	Hulled gain yield	No. of panicles	No. of spikelet/ panicle	No. of spikelet	Ripened grains^a^	1000 grain weight	Sink capacity	Sink filling
					g m^-2^	g m^-2^	m^-2^		m^-2^	%	g	g m^-2^	%
2012													
	H193	May 24	June 22	Aug 30	924	881	294	152	44756	86	22.8	1019	91
	MOMI	May 15	June 22	Aug 30	844	809	254	167	42417	71	26.9	1144	74
Cultivar effect (*p*)					*	*	**	**		***	***	ns	***
2013													
	H193	May 31	June 20	Aug 30	775	720	315	143	45188	68	23.6	1066	73
	MOMI	May 17	June 20	Aug 30	637	546	266	165	43812	48	25.9	1136	56
Cultivar effect (*p*)					***	**	*	**		**	***	ns	**
2014													
	H193	May 30	June 18	Aug 30	837	820	261	147	38399	88	24.2	928	90
	MOMI	May 20	June 18	Aug 30	852	816	256	157	40126	75	27.2	1088	78
Cultivar effect (*p*)					ns	ns	ns	ns		**	***	*	**
Cultivar mean													
	H193	May 28	June 20	Aug 30	845	807	290	148	42809	81	23.5	1005	85
	MOMI	May 17	June 20	Aug 30	778	724	258	163	42115	65	26.7	1122	70
Cultivar effect (*p*)					***	***	***	***	ns	***	***	***	***
Year mean													
2012		May 20	June 22	Aug 30	884a	845a	274ab	160	43737a	79a	24.9b	1081ab	82a
2013		May 24	June 20	Aug 30	706c	633b	291 a	154	44768a	58b	24.8b	1101 a	64b
2014		May 25	June 18	Aug 30	844b	818a	258 b	152	39274b	82a	25.7a	1108 b	84a
Year effect (*p*)					***	***	**	ns	**	***	***	*	***
Cultivar x year (*p*)					***	**	*	ns	ns	ns	***	ns	ns

Significant difference:

*p = 0.05

**p = 0.01

***p = 0.001

### NSC and starch accumulation in stems

**Fig 1:**
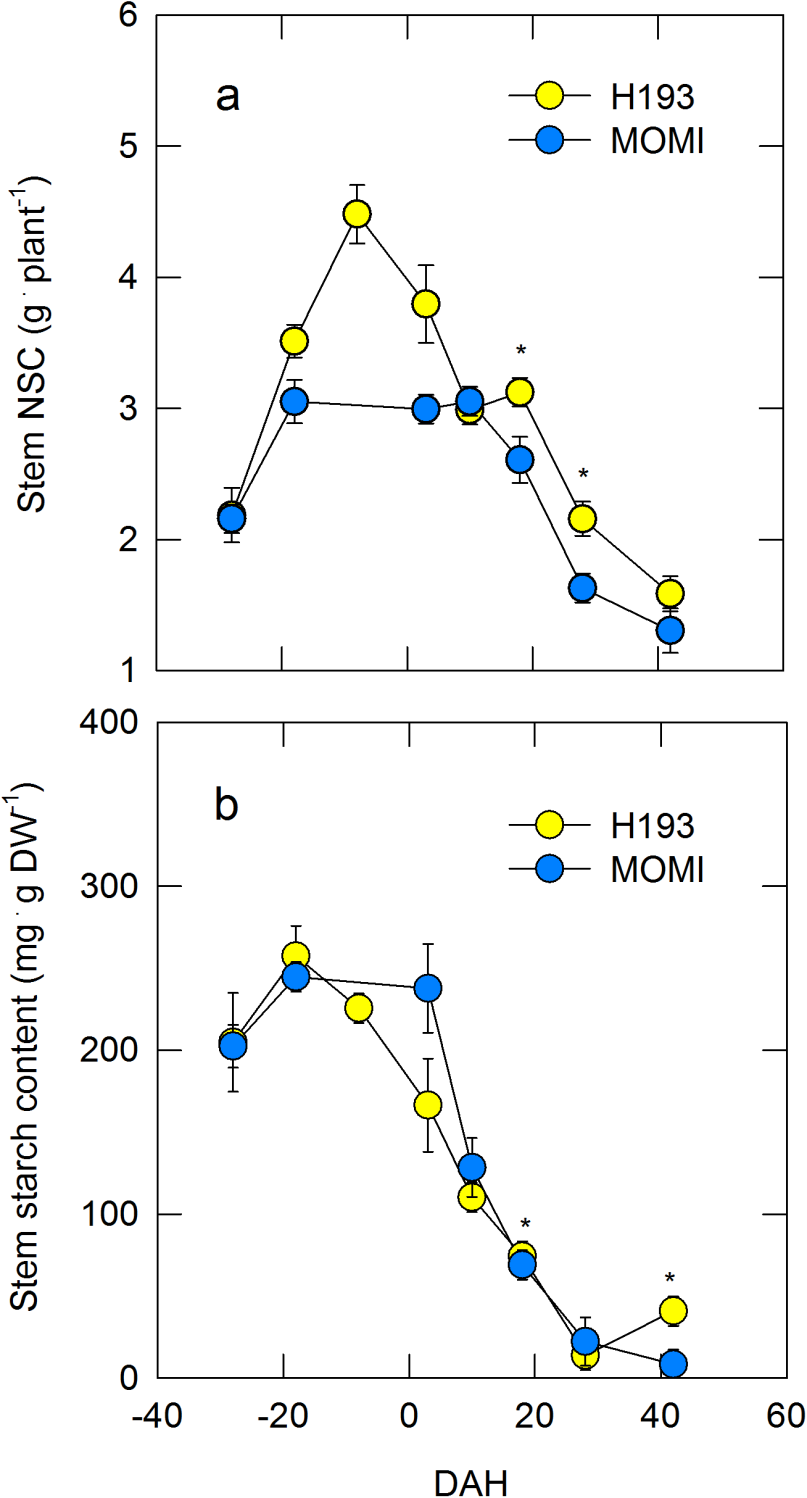
Time course of changes in nonstructural carbohydrate per plant (a) and starch content (b) in the stems (culms and leaf sheaths) of two high-yielding rice cultivars during development in field-grown plants in 2013. Yellow and light blue circles indicate H193 and MOMI, respectively. Data in a and b are the mean ± SEs of *n* = 3. Significance at the 0.05 probability levels is indicated with *.

**Fig 2:**
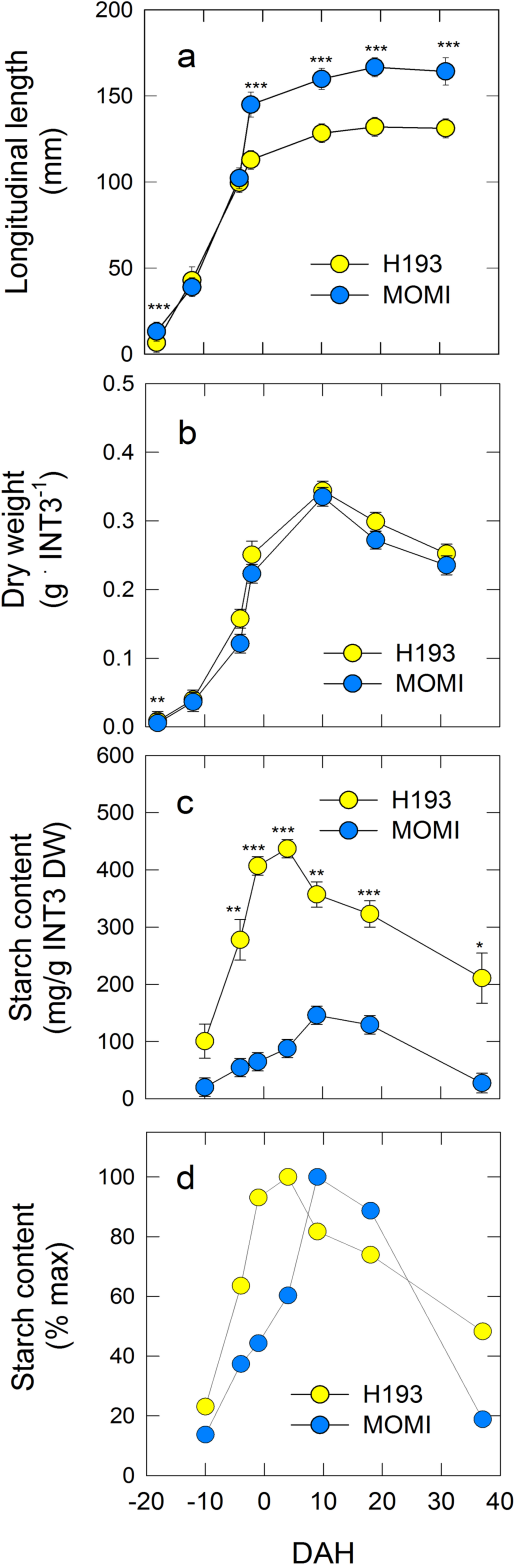
Time course of changes in longitudinal length (a), dry weight (b), starch content (c), and relative value of starch content (d) in developing INT3 in two high-yielding rice cultivars in 2014. Yellow and light blue circles indicate H193 and MOMI, respectively. Data in a and b-d are the mean ± SEs (*n* = 30) and (*n* = 3) of field-grown plants, respectively. Significance at the 0.05, 0.01 and 0.001 probability levels is indicated with *, **, and ***, respectively. Note that there were substantial varietal differences in terms of both starch content (c) and the timing of starch accumulation (d).

There was a significant difference in the seasonal pattern of stem (culms and leaf sheaths) NSC accumulation (Fig 1A). Stem NSC in H193 was greater than that in MOMI, particularly during the pre-heading stage. NSCs in H193 increased until -8 DAH, reaching 4.4 g plant^-1^, and thereafter decreased rapidly to 1.5 g plant^-1^ at 40 DAH. Stem NSC in MOMI was 3 g plant^-1^ at the heading stage, remained constant until 10 DAH, and then declined until 40 DAH (Fig 1A). Starch content in two HYVs was in the same range, 0–250 mg g DW^-1^. Starch in H193 began to gradually decrease from -18 DAH, whereas starch in MOMI appeared to decline after 4 DAH (Fig 1B). Because internode 3 (INT3) in the stems was an organ that most accumulated NSC at full-heading (S2 Fig), we focused on carbohydrate metabolism in INT3 for further analysis. The longitudinal length of INT3 in MOMI was greater than that in H193, but growth of INT3 itself was synchronized under field conditions (Fig 2A), as was the heading date (Table 1). Changes in INT3 dry weight exhibited similar trends in the two HYVs (Fig 2B), but starch content in H193 was considerably higher than that in MOMI (Fig 2C). Furthermore, the initiation of starch degradation in H193 was 5 days earlier than that in MOMI (Fig 2D).

### Plant-water relations

**Fig 3:**
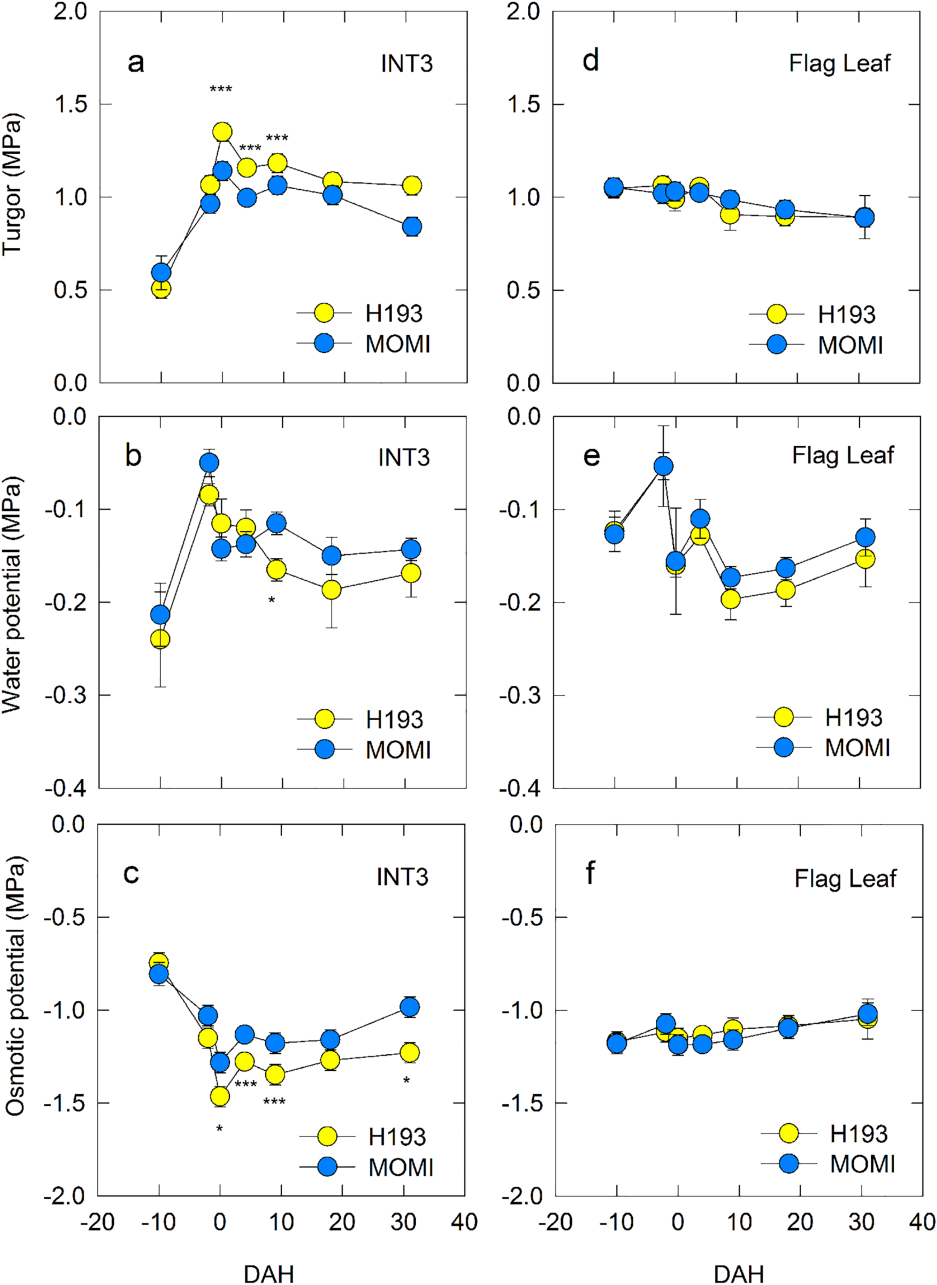
Changes in turgor pressure (a,d), water potential (b,e), and osmotic potential (c,f) of INT3 (a-c) and flag leaf (d-f) during development. Yellow and light blue circles indicate H193 and MOMI, respectively. Data are mean ± SEs for 3–4 plants from three independently repeated experimental plots in 2014. Significance at the 0.05 and 0.001 probability levels is indicated by * and ***, respectively.

The water status of INT3 changed remarkably from -10 DAH to 32 DAH (Fig 3A–3C). After the turgor pressure of H193 INT3 increased from 0.5 MPa to 1.4 MPa while approaching the heading stage, turgor slightly decreased until 18 DAH, and was stable at 1.1 MPa through 32 DAH (Fig 3A). A similar trend was observed in turgor pressure of MOMI INT3, but the level of turgor pressure was lower than that of H193 (Fig 3A). After the water potential of INT3 increased from -10 to -2 DAH reaching -0.08 MPa, water potential gradually declined until 30 DAH (Fig 3B). Furthermore, the water potential of H193 INT3 tended to be lower than that of MOMI (Fig 3B), indicating that more osmotically active solutes accumulated in the apoplastic space in H193 during post-heading stage. In addition, the decline in osmotic potential was observed while approaching the heading stage, and then gradually increased after heading in both varieties (Fig 3C). Turgor pressure of the flag leaf gradually declined over the development (Fig 3D), and turgor pressure of the H193 flag leaf after heading was lower than that of INT3 (Fig 3A and 3D). Leaf water potential ranged between -0.2 and -0.05 MPa (Fig 3E). Leaf osmotic potential increased slightly during development (Fig 3F). Overall, no varietal difference was observed in the water status of flag leaf throughout development (Fig 3D–3F).

### Prefixed ^13^C analysis

**Fig 4:**
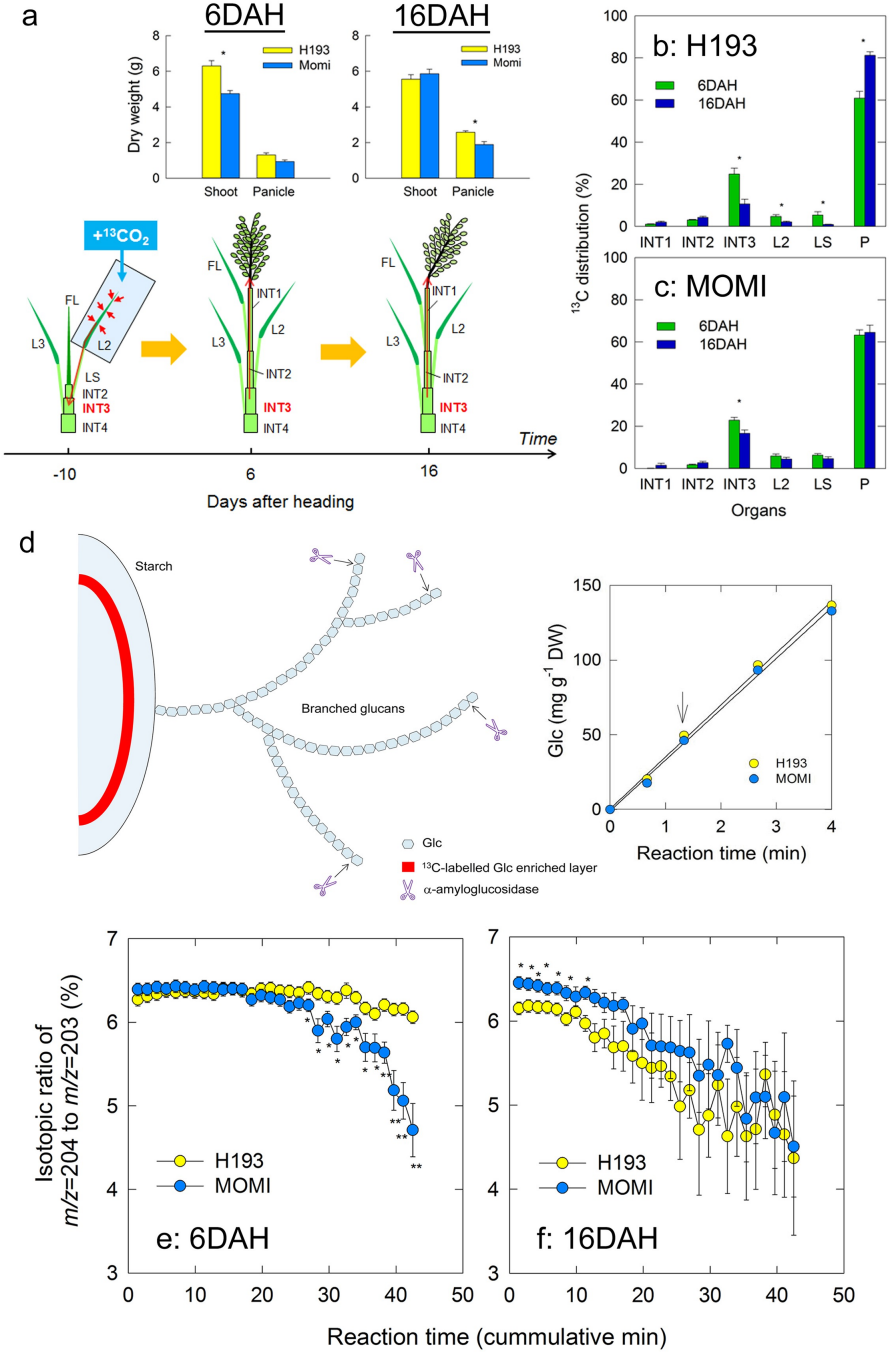
a, diagram of the prefixed ^13^C assay, and shoot (including a panicle) and panicle dry weights of H193 and MOMI collected at 6 and 16 DAH that exhibited a distinct varietal difference for the peak of NSC and starch accumulation (Figs 1 and 2). The field-grown plants in 2013 were labeled with ^13^CO_2_ from the 2nd leaf (L2) at -10 DAH (see Methods). b and c, changes in ^13^C distribution in each organ including the first internode (INT1), the second internode (INT2), the third internode (INT3), the 2nd leaf blade (L2), the 2nd leaf sheath (LS), and panicle (P) in H193 (b) and MOMI (c) collected at 6 (green) and 16 DAH (blue). d, a putative diagram of starch granules with branched glucan chains extracted from INT3 that has ^13^C-labelled Glc enriched layer (red zone). α-amyloglucosidase (scissor) is capable of hydrolyzing the α-D-(1–4), the α-D-(1–6), and the α-D-(1–3) glucosidic bonds of oligosaccharides to convert starch to Glc. Glc content produced by the reaction of α-amyloglucosidase with each HYV starch as a function of the reaction time. The regression line between the reaction time (*x*) and Glc content (*y*) in H193 and MOMI was *y* = 34.77*x* +0.398 with *r*^2^ = 0.99 (*p*<0.001) and *y* = 34.04*x* – 1.01 with *r*^2^ = 0.99 (*p*<0.001), respectively, showing no significant varietal difference at least <4min. The arrow indicates the set reaction time, 1.42 min. In e and f, ^13^C/^12^C isotope ratio of sodiated Glc yielded from INT3 starch at 6 DAH (e) and 16 DAH (f) as a function of cumulative reaction time. A putative ^13^C-enriched layer in the starch granules in each day was shown in red. The trend of isotopic ratio became opposite between 6 and 16 DAH among cultivars, indicating that starch degradation progressively occurred in H193 between 6–16 DAH as ^13^C distribution in panicle increased (Fig 4B). In a, d, e and f, yellow and light blue symbols indicate H193 and MOMI, respectively. Each point in a-c and e-f is the mean ± SEs (*n* = 3–5) of field-grown plants in 2014. Significance at the 0.05 and 0.01 probability levels is indicated with * and **, respectively.

At -10 DAH, when the INT3 starch content in both varieties was relatively low with no significant difference (Fig 2D), ^13^C was pre-fixed, and then the ^13^C distribution in plants collected at 6 and 16 DAH was analysed (Fig 4A). Significant varietal differences in dry weight per panicle were observed at 16 DAH (Fig 4A). The ^13^C distribution percentage in INT3 of both varieties was approximately 25% at 6 DAH (Fig 4B and 4C). A significant reduction in ^13^C in INT3 at 16 DAH was observed in both varieties; however, an increase in ^13^C distribution in panicles between 6 and 16 DAH was only observed in H193 (Fig 4B and 4C). In addition, reduction of the ^13^C distribution in the 2nd leaf blade (L2) and leaf sheath (LS) was observed only in H193 after 10 days (Fig 4B). To trace ^13^C-labelled Glc synthesized in starch granules in INT3 during pre-heading, we then extracted ethanol-insoluble fraction from the tissues each day, and analysed the ^13^C-labelled Glc enriched layer embedded in starch granules by combining mass spectrometer analysis with an enzymatic reaction (Fig 4D), according to the previous study [31]. At 4 DAH, when starch content in H193 reached a maximum, but MOMI continued accumulating starch (Fig 2D), the isotopic ratio of the most abundant sodiated C_5_^13^CH_12_O_6_ (*m/z* = 204) to sodiated C_6_H_12_O_6_ (*m/z* = 203) in MOMI at 6 DAH significantly declined over the cumulative reaction time until 20 min, whereas that of H193 remained high and stable above 6% (Fig 4E). At 16 DAH, when the NSC in MOMI began to decline (Fig 1), the isotopic ratio in H193 was significantly lower than in MOMI, even with a short reaction time (Fig 4F). Significant differences were observed for the shallow depth between the two varieties (Fig 4F), indicating that metabolic switching from starch synthesis to degradation in stems occurred much earlier in H193 than MOMI, even though H193 had greater starch content at full-heading (see Fig 2C).

### Gene expression in the 3rd internode

**Fig 5:**
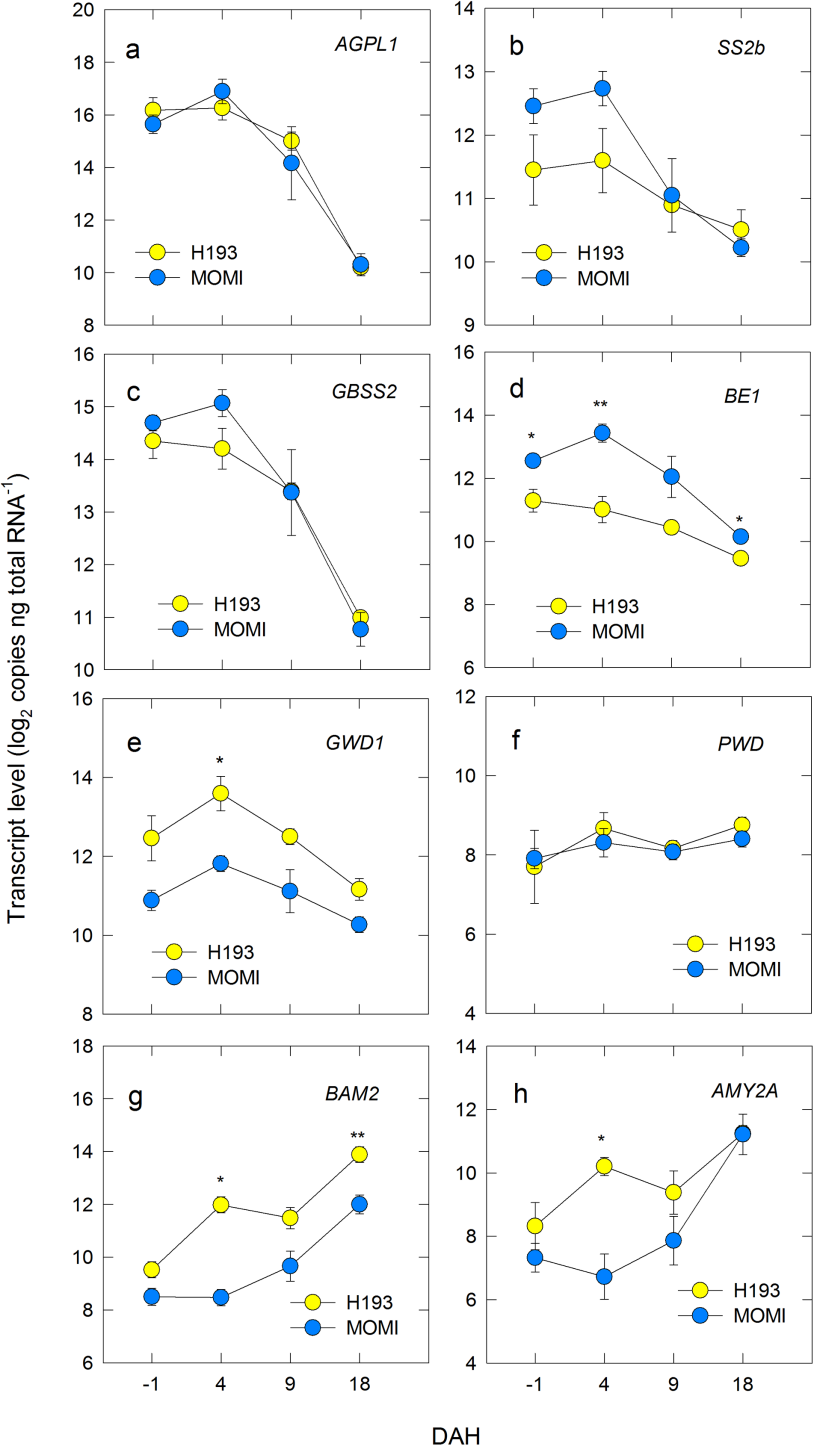
Changes in transcript levels of AGPL1 (a), SS2b (b), GBSS2 (c), BE1 (d), GWD1 (e), PWD (f), BAM2 (g), and AMY2A (h), in the mature region of INT3 in H193 and MOMI during development. Yellow and light blue symbols indicate H193 and MOMI, respectively. The data indicate that expression of *BE1* in MOMI was higher than H193. In contrast, *GWD1* was greater in H193 than MOMI, but not for *PWD*, and starch degradation-related genes, *BAM2* and *AMY2A* in H193 preceded to MOMI. Data for gene expression levels are the mean ± SEs (*n* = 3) from the pooled 3 field-grown plants collected from three independently repeated experimental plots in 2014. Significance at the 0.01 and 0.05 probability levels is indicated by ** and *, respectively.

**Fig 6:**
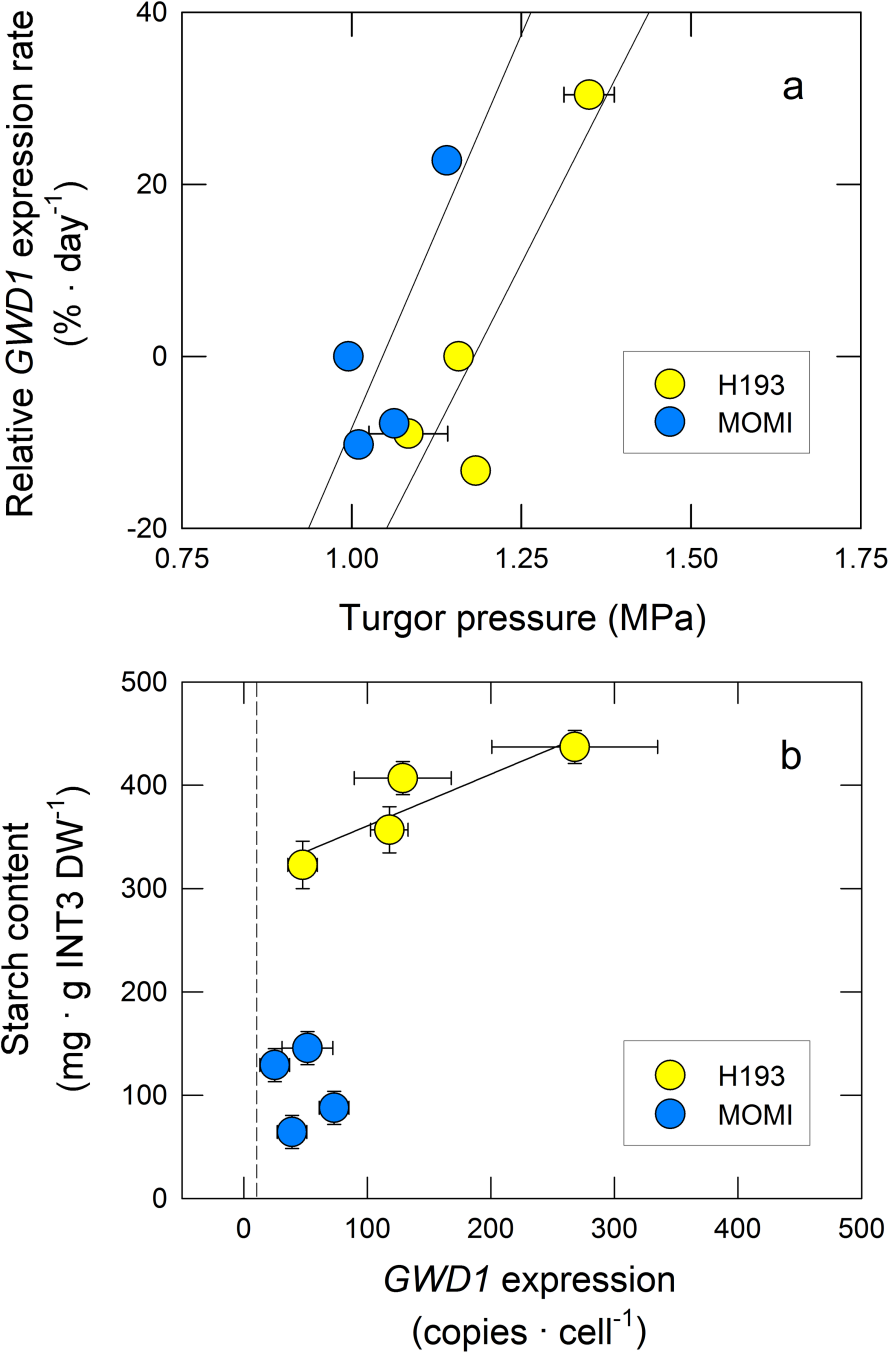
Relative *GWD1* expression rate as a function of turgor pressure in the mature region of INT3 in H193 and MOMI (a). Starch content plotted against *GWD1* expression level estimated per cell in H193 and MOMI (b). The solid lines indicate regression lines. Yellow and light blue symbols indicate H193 and MOMI, respectively. The linear regression between turgor pressure (collected between 0 and 18 DAH, see Fig 3A) and relative *GWD1* expression rate in H193 and MOMI was y = 155.06x-182.99 (*r*^2^ = 0.78) and y = 182.87x-191.17 (*r*^2^ = 0.63), respectively. The dashed line in b indicates 10 copies per cell, which corresponds the border of biologically insignificant basal level of expression. The linear regression between starch content and *GWD1* expression level per cell in H193 was y = 0.50x +310.66 (*r*^2^ = 0.82).

To investigate changes in gene expression involved in the starch metabolism in INT3, we conducted genomic DNA-based absolute quantification [30]. Marked differences in the transcript level, as well as pattern, were found in both starch synthesis and degradation between MOMI and H193. Gene expression of starch synthesis-related enzymes in MOMI peaked at 4 DAH, later than those in H193 (Fig 5A–5D). Transcript levels of neither the ADP-Glc pyrophosphorylase (AGPase) large subunit (*AGPL1*) (Fig 5A) nor the AGPase small subunit (*AGPS1*) (S3 Table) significantly differed between the varieties. Gene expression of starch synthase IIb (*SS2b*) and granule-bound starch synthase (*GBSS2*) tended to be higher in MOMI than in H193 until 4 DAH (Fig 5B and 5C). Additionally, gene expression levels of branching enzyme (*BE1*) in MOMI were much greater than those in H193 (Fig 5D). In contrast, transcript levels of α-glucan, water dikinase (*GWD1*) in H193 were much higher than those in MOMI throughout the duration of the analysis (Fig 5E), whereas gene expression of another glucan phosphorylating enzyme, phosphoglucan, water dikinase (PWD)-like protein did not differ between the varieties (Fig 5F). Because peaks in turgor pressure was observed prior to the increase in *GWD1* expression in both HYVs, we analysed the relationship between turgor pressure and changes in gene expression. When the relative *GWD1* expression rate was plotted against turgor pressure, there was a linear and positive correlation in each HYV (Fig 6A). These correlations were shown to be parallel with approximately 0.2 MPa of turgor pressure difference (Fig 6A), indicating that turgor pressure was closely associated with changes in *GWD1* expression in both HYVs. When starch content was plotted against the level of *GWD1* expression per cell, a linear and positive correlation was observed in H193, but not for MOMI (Fig 6B). Up-regulation in gene expressions of α- and β-amylase was also observed in both HYVs, but it was substantially delayed in MOMI (Fig 5G and 5H). Expression of a major gene in the leaf sheath [18], *AMY2A* encoding α-amylase 2A, and *BAM2* encoding β-amylase 2, reported as a major gene expressed in stems at the post-heading stage [32] (S4 Table), rapidly increased in H193 during -1 to 4 DAH, whereas those of MOMI began to increase after 4 DAH (Fig 5G and 5H). In addition, transcript levels of both amylase genes in MOMI were significantly lower than those in H193 (Fig 5G and 5H). Unlike *GWD1* expression, there was no clear linear relations between turgor pressure and the rate of *BAM2* or *AMY2A* expression (S3 Table), suggesting that these amylase genes were unlikely to be associated with turgor pressure. Gene expression patterns of three sugar transporters, maltose exporter (*MEX1*), plastidial glucose transporter (*pGlcT*), and sucrose transporter (*SUT1*) were similar to those of *GBSS2* and *SS2b*, but no significant differences were observed between the varieties (S3 Table).

## Discussion

In this work, we hypothesized that turgor pressure affects nuclear gene expression. Our results show that an increase in expression of the key glucan phosphorylating enzyme-encoding gene, *GWD1* was closely associated with turgor pressure in rice stems (Fig 6A). It has been also shown that subspecies differences in the pattern of stem NSC between *indica-* and *japonica*-dominant HYVs could have originated from varietal differences of turgor-associated starch phosphorylation in stem storage parenchyma cells. Unlike flag leaves, the water status of INT3, which was the largest NSC storage organ (see S2 Fig), was dynamic from pre-heading through ripening, displaying varietal differences (Fig 3). An increase in turgor pressure coincident with solute accumulation observed at heading was larger in H193 than in MOMI (Fig 3A). Dynamic pre-fixed ^13^C tracer analysis proposed that starch synthesis followed by rapid starch degradation in stems was attributable to the rapid decline in NSC in H193 (Fig 4). The absolute quantitative PCR analysis (Fig 5) revealed that there were remarkable varietal differences in the expression of starch synthesis- and degradation-related genes, in particular the contrasting expression patterns between *BE1* and *GWD1* at full-heading (Fig 5). Increasing *GWD1* expression, that plays a crucial role at the initial step of starch breakdown (Figs 5 and 6B), appeared to precede the upregulation of both α- and β-amylase-encoding genes in both HYVs, consistent with the previous studies [19, 20, 33, 34]. Taken together, it is concluded that turgor-associated starch phosphorylation in cells is responsible for the metabolism that leads to starch degradation, which would facilitate sugar transport to panicles from the stems. This explains subspecies differences in stem NSC partitioning that determines the grain-filling ability between *indica*- and *japonica*-dominant HYVs [17].

**Fig 7:**
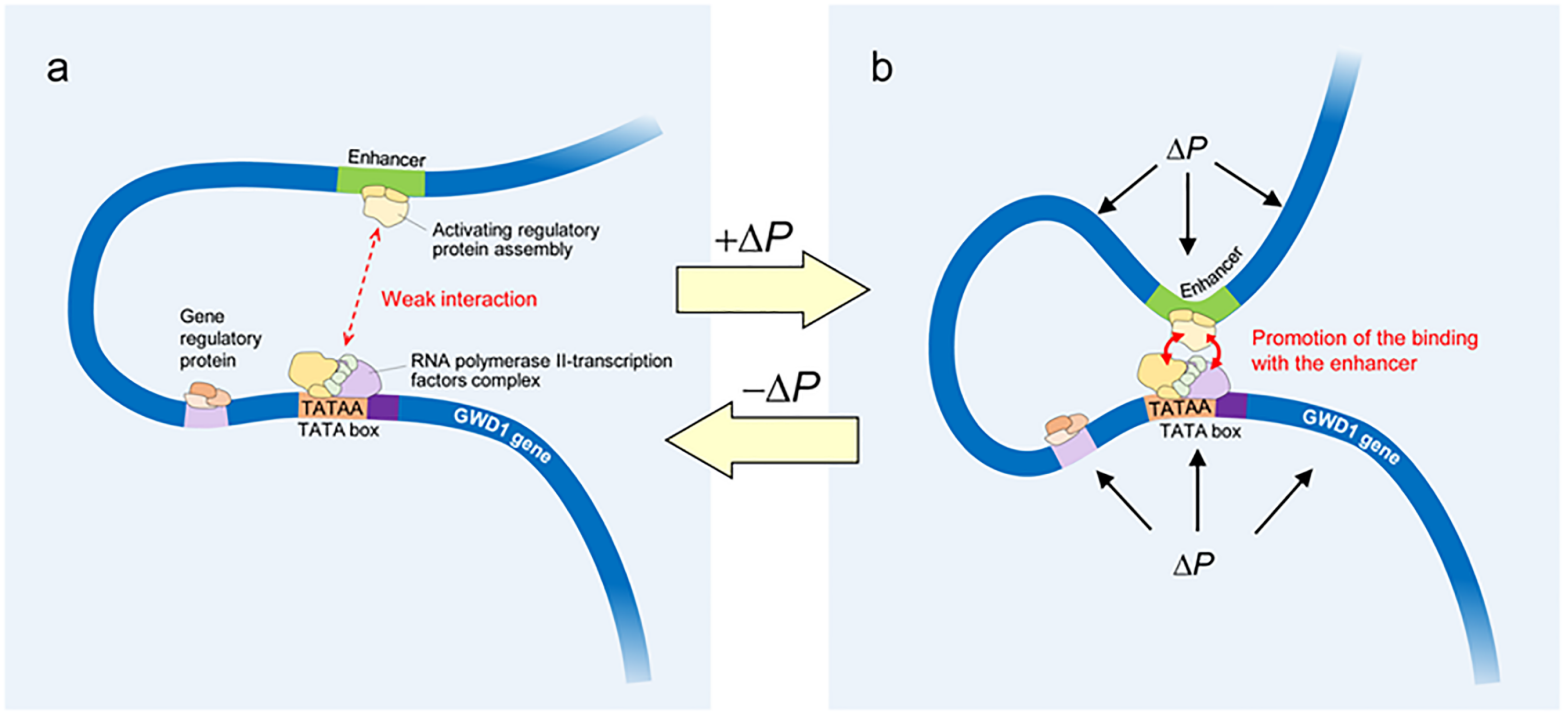
Schematic diagrams of possible turgor-induced nuclear gene expression. In a, RNA polymerase II binds to the promoter region, together with transcription factors, and the complex interacts with an activator attached to the enhancer to initiate gene expression. When Δ*P* is applied, it is speculated that Δ*P* might be perceived by both the promoter region of *GWD1* gene and the enhancer region, resulting in the promotion of multiple protein-DNA complexes binding (RNA polymerase II-transcription factors complex and the activating regulatory protein assembly) to increase gene expression (b). It is possible that these responses may occur reversibly in accordance with the changes in turgor pressure.

To date, there has been some evidence for turgor-responsive gene expressions [35]. Interestingly, there has been a consistent finding that down-regulation of turgor pressure is closely associated with substantial changes in gene expressions in several physiological processes, such as dehydration [35, 36] and fruit softening [4]. Conversely, the results of the present work represent that *GWD1* expression might be influenced by an upregulation of turgor pressure (Fig 6A). To initiate gene expression, RNA polymerase II-transcription factors complex is required to interact with the enhancer [21]. During the process, water molecules should participate in the recognition of the protein-DNA complexes [22–24], as well as other macromolecules acting at translational level. When the size of changes in turgor pressure (Δ*P*) is less than diurnal oscillation or a threshold, as described below (Fig 7A), it is presumed that the binding might be weak. When Δ*P* is applied, it is speculated that the structure of the DNA double helix might be altered, resulting in promoting the binding of multiple protein-DNA complexes to increase gene expression (Fig 7B). Whether Δ*P* is directly or indirectly transmitted to the nucleus remains elusive, however, it is reasonably assumed that Δ*P* would be perceived by multiple sites during nuclear gene expression (Fig 7B). In addition, this response may reversibly occur depending on the size of Δ*P*. This work has proposed the possibility of turgor-associated transcriptional regulation of *GWD1* expression in rice plants, although it remains uncertain how turgor pressure induces favourable conformational changes of the complexes at molecular level. This will need to be addressed in future research.

Manipulation of NSC is generally recognized as a way to stabilize and increase productivity in most crops [14]. In rice, the apparent contribution of stem NSC to grain yield is approximately 30% under normal conditions [12]. Recently, it has been reported that there were subspecies differences in NSC accumulation patterns in the stems [17]. Similarly, subspecies differences in starch accumulation patterns have been reported in leaf sheaths between the *japonica*-dominant variety, Nipponbare, and the *indica*-dominant variety, Takanari [18]. When varietal differences in NSC partitioning at the post-heading stage was studied, it would be ideal to synchronize the heading dates to carry out the research, so that the effects of environmental fluctuations during pre- through post-heading stages could be minimized. However, it seems unlikely that this issue has received much attention [17, 18], presumably because of the inherent difficulties in such experiments. In this study, heading dates of each field-grown HYV were synchronized successfully, and hence any age-related difference between the two HYVs examined were minimized during the stages. Our data also showed that similar varietal differences were exhibited in both starch and NSC accumulations (Fig 1). Furthermore, prefixed ^13^C analysis showed that starch synthesis and breakdown in H193 stems both occurred more rapidly than those in MOMI. Also, an absolute quantitative PCR analysis [30] allowed us to precisely determine varietal differences at the transcriptional level. This indicated that an increase in starch phosphorylation by GWD1, but with not for PWD, occurred prior to starch breakdown (Figs 2 and 5), consistent with the results of the ^13^C tracer assay. Therefore, it is strongly suggested that there is a substantial subspecies difference in starch phosphorylation occurring in amyloplasts.

Over the past decade, our knowledge regarding the regulation of starch metabolism has increased [20, 34]. Starch breakdown was shown to be facilitated by phosphorylation of glucan initiated by the action of α-glucan, water dikinase, which leads to an increase in a series of amylase activity [19]. Our results also showed clear differences between MOMI and H193 in the expression of starch metabolism-related genes in INT3 (Fig 5). The transcript levels of *GWD1*, *AMY2A*, and *BAM*2 in MOMI appeared to be low, and increasing amylase-related gene expression was delayed approximately 10 days compared with that of H193 (Fig 5E, 5G and 5H). Expression of these amylase-related genes changed in parallel with a decrease in starch content (Figs 2C, 2D and 5), consistent with observations reported for the leaf sheath [18]. It has been reported that a series of starch phosphorylation by GWD and PWD is required for normal mobilization of transitory starch in leaves [25, 26]. A rice mutant defective in *GWD1* expression was used to reveal that rice GWD1 also phosphorylated glucans, and is involved in starch degradation in leaves with a diurnal rhythm [37]. Although the mutant with hyper-accumulated leaf starch grew normally during the vegetative stage, it produced lower grain yield than the wild-type [37], suggesting that GWD1 in rice stems might affect sugar transport to panicles during the reproductive stage. Given that varietal differences on *GWD1* and *BE1* expression have been observed to contrast, there might be some interaction between GWD and BE at the surface of granules that alters physicochemical properties of starch during the post-heading stage.

**Fig 8:**
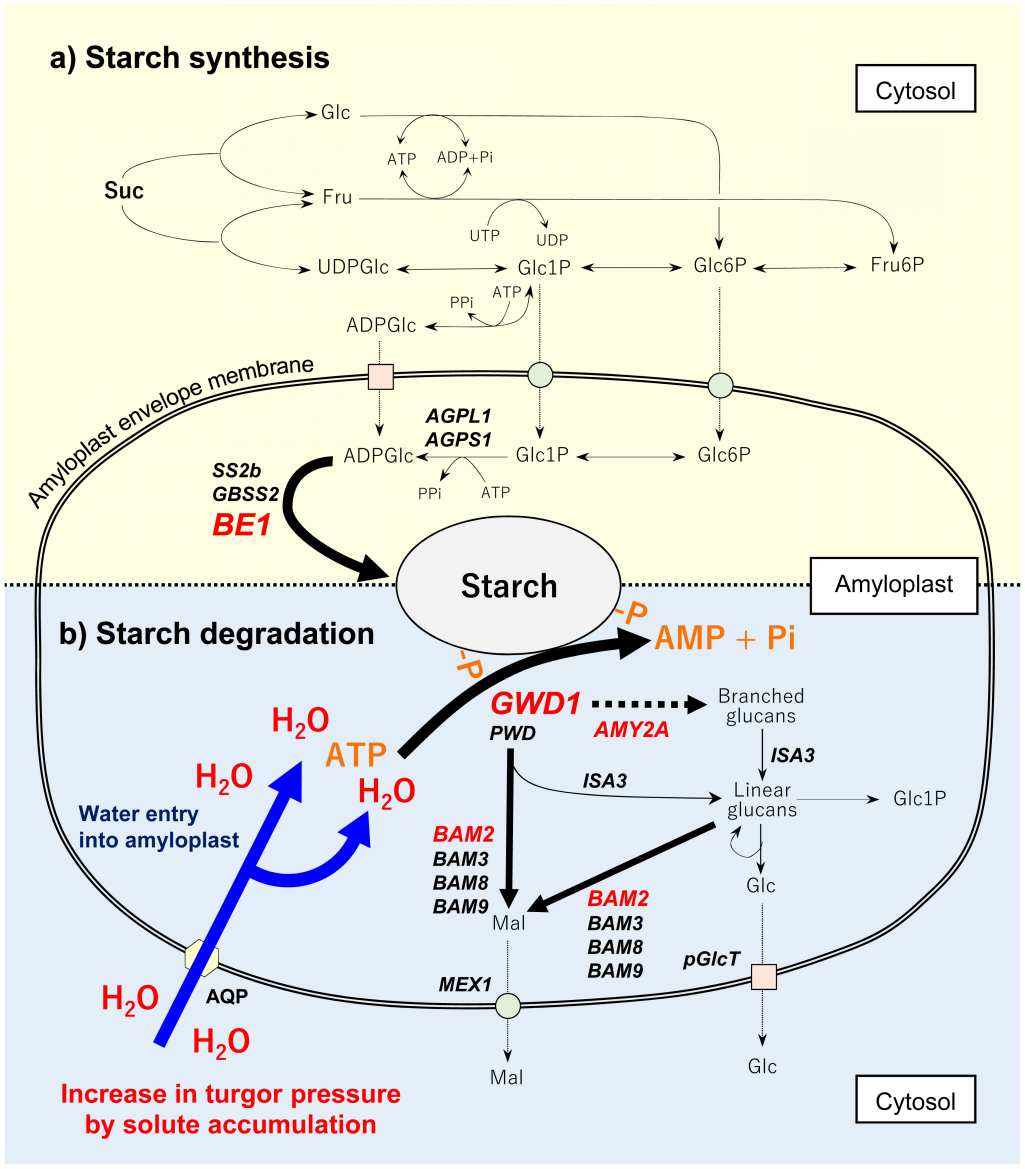
Putative pathway of starch synthesis (a) and degradation (b) in *Oryza sativa* stems at early ripening stage. Italicized indicate genes analyzed in this work (see S3 Table). For genes, *BE1*, *GWD1*, *AMY2A*, and *BAM2* shown in red, there were significant varietal difference among two HYVs. Water entry into amyloplast (shown in blue) caused by an increase in turgor pressure may be required for the initiation of starch phosphorylation by the activation of GWD, but not for PWD, followed by the elevated amylase activity to degrade starch. In b, putative water-transporting proteins (AQP) localized on the amyloplasts envelope membrane is also indicated.

Regarding the varietal differences in starch degradation, our data also indicated that starch degradation in INT3 of H193 and MOMI began from 4 and 9 DAH, respectively (Fig 2D). The same stem coincidently displayed a rapid increase in turgor pressure leading up to heading, which was mainly caused by accumulating osmotically active solutes in the cells (Fig 3A and 3C). Moreover, a relatively large varietal difference in osmotic potential (ca. 0.2 MPa) has been detected at heading by using isopiestic psychrometers (Fig 3C). Clearly, these changes in cell water status occurred prior to starch degradation (Fig 2D) induced by the up-regulated expression of *GWD1* observed up to 4 DAH, followed by the increase in expression of several amylase-related genes (Fig 5 and S3 Table). In addition to nucleus, the volume of plastids is also known to be osmotically responsive [25]. When starch breakdown occurs in amyloplasts, an increase in the single glucan chains on the surface of starch granules by starch phosphorylation with the activation of GWD1 should allow other starch-related enzymes easily access to degrade the chains, resulting in rapid starch degradation [19, 20, 34], as observed in H193 (see Fig 4). The activity of GWD1 in amyloplasts requires both ATP and a water molecule as substrates for the solubility of starch to be partially increased in the amyloplasts [19]. This indicates that, prior to the reaction a certain amount of water is needed at the reaction site on the surface of the granules in the amyloplasts, as illustrated in Fig 8. If this is the case, water entry over the amyloplast envelope associated with a substantial increase in turgor pressure is likely the precondition for starch phosphorylation in the storage parenchyma cells.

Several studies have also provided evidence that some water channel proteins localize in chloroplast envelope in several plants (e.g., ref. [38] for PIP1 in tobacco leaves). To our knowledge, there is no direct evidence of the presence of water channel proteins in rice amyloplast envelope. However, it is reasonable to assume that the water flow into amyloplast would occur through the proteins (see Fig 8). Our data has raised the new possibility that turgor pressure plays a regulatory role in starch phosphorylation at both transcriptional and metabolic levels as a primary event in carbohydrate partitioning in the stems (Figs 6–8). If starch phosphorylation were turgor-responsive, then it is reasonable to assume that turgor pressure increases leading up to heading might have a threshold above which *GWD1* expression would be significant, as proposed in Fig 7. This is what we observed (see Fig 6). Hence, this would explain the varietal difference for the level of *GWD1* expressions (Figs 5E and 6), which caused the lag time of starch degradation between subspecies displayed as the different NSC patterns (Fig 1).

Besides the involvement of turgor pressure in cell enlargement [1–3] and fruit softening [4], the results of this study suggested another possibility for turgor regulation of NSC partitioning as a high-yielding trait in rice plants. It has been postulated that in rice leaf sheaths phloem unloading into storage parenchyma cells would occur symplastically [39]; however, how sucrose is transported from the cells to phloem is unclear [14]. Considering that plasmodesmal conductivity is pressure-sensitive [27], lowering of turgor pressure at the post-heading stage, as we observed (Fig 3A), may suggest another functionally important role in NSC partitioning. Because the water status measurement in INT3, completely covered with a leaf sheath, was conducted in the early morning, transpiration from the stem could be negligible, which indicates there was essentially no matric potential in apoplastic space. We did not detect any subspecies difference in the sugar transporters including *SUT1*, at least at the transcription level (S3 Table), although the observed lowering of water potential could be explained by apoplastic sugar accumulation, as proposed previously [40]. The lowering of water potential by apoplastic solute accumulation is likely to contribute to a decline in turgor pressure during the post-heading stage (Fig 3), as reported in other plants, such as sugarcane [41] and grape fruits [42, 43]. As previously stated [14, 40], it is possible that plasmodesmatal closure and switching to the apoplastic pathway both occur during the NSC partitioning process. In this view, there may be subspecies differences in plasmodesmata density or its mechanism of control, in addition to the pathway change for phloem loading in the stems. However, these possibilities await future investigation.

Finally, it is recognized that two subspecies, *japonica* and *indica*, are descended from different ancestors [44]. It is also known that *japonica* strains are closely related to perennial strains, whereas *indica* strains are annual-related [45]. Re-distribution of NSC at the later stage during ripening [13, 46], as well as ratooning is typical features of *japonica*-dominant rice [47]. The *japonica*-dominant HYV, Momiroman, used in this work, was established from a cross of a New Plant Type with large panicles, IR65598-112-2, and a *japonica*-dominant HYV, Saikai 203 (later named Mizuhochikara), and backcrossed with the line Saikai 203 three times [48]. Taking consideration that IR65598-112-2 was bred from two tropical *japonica* rice strains, often showing similar low grain-filling rates [15], it may be that the same mechanism exists in other cultivars with relatively large spikelets, such as *japonica*-dominant HYVs and NPT rice. Further analysis will contribute to the development of selection criteria for breeding programs in terms of NSC partitioning at the post-heading stage.

In conclusion, we showed that the observed subspecies difference in stem NSC partitioning, that determines grain-filling ability among *indica*- and *japonica*-dominant HYVs, could have originated from turgor-associated regulatory mechanisms in starch phosphorylation in stem storage parenchyma cells. Thus, this new aspect may provide an effective way for increasing the yield potential in the certain cropping season(s), when plants rely on temporarily stored NSC in the stem. Considering rice production under climate change, subspecies difference in NSC partitioning under heat stress conditions will also need to be addressed. Future studies will contribute to the enhancement of rice production by increasing yield potential under climate change.

## Materials and methods

### Plant materials

Two rice varieties, H193 and MOMI, were grown in field experiments conducted on Gray Lowland soil (Typic Endoaquepts) at the Kyushu Okinawa Agricultural Research Center (33°12′19″N, 130°29′23″E, altitude 10 m), Chikugo, Fukuoka, Japan in 2012–2014. The daily mean temperature and radiation at each stage, recorded by a weather station near the experimental field, are shown in S1 Table. The experimental plots in 2012, and 2013 and 2014 were 13.5 and 16.5 m^2^, respectively (nine rows × 5.0 m length in 2012, and 11 rows × 5.0 m in 2013 and 2014). A randomized complete block design with three replicates was used each year. Two HYVs seedlings, 29 days old for H193 and 38 days old for MOMI in 2012, 24 days old for H193 and 34 days old for MOMI in 2013, and 29 days old for H193 and 38 days old for MOMI in 2014 were transplanted on the same day each year (see Table 1) to synchronize the full-heading dates across the three years. The full-heading date was defined as the date when approximately 80% of panicles in a canopy had emerged. Planting density was 20.8 hills per square meter (hill spacing of 0.3 × 0.16 m), with three seedlings per hill. Each year, seven days before planting, 18 g N m^-2^, 10.8 g P m^-2^, and 10.8 g K m^-2^ in a slow-release fertilizer, which was composed of the mixture of LP20 and LPSS100, was applied by hand as basal dressing and the fertilizer was incorporated into the soil by puddling. The experimental plot was well managed, without stress from weeds, disease, insect pests, nutrients, or other factors. On each NSC sampling date, two hills per plot were dug, the roots were cut off, and the samples were washed to remove soil. Dry weight (DW) of the stem (culm and leaf sheath) was determined as described previously [49] and used for the following NSC assay after grinding into a fine powder.

### Yield and yield component determination

Yield and yield components were measured from 48 hills per plot that were harvested at maturity. The yield and 1,000-grain weight were recorded as the ripened grain (thickness ≥ 1.7 mm) weight of brown rice adjusted to 0.15 g g^-1^ grain water content. The percentage of ripened grains was calculated as 100 × (ripened grains) × (total spikelet number)^-1^. Sink capacity was defined as single grain weight multiplied by the number of spikelets per square meter. Sink filling rate was calculated as the hulled grain yield divided by sink capacity. Spikelets in each portion were further classified as in a previous study [50], and within-panicle ripened grains were evaluated (see S2 Table).

### Non-structural carbohydrate determination

The NSC concentration was determined according to the gravimetric method [51] with correction for water content [49].

### Prefixed ^13^C isotope tracer analysis and starch assay

The ^13^C feeding experiments were conducted in field-grown plants at -10 DAH in 2013 for the pre-fixed ^13^C isotope analysis focusing on the third internode (INT3) (Fig 4A). The second leaf was exposed to ^13^CO_2_. ^13^CO_2_ was applied by gently enclosing the leaf inside a 1.27 L polyester gas sampling bag with a plastic container containing 0.5 g of Ba^13^CO_3_ (99 atom% ^13^C) and ^13^CO_2_ was generated by injecting 2 mL lactic acid from the outside of the bag. The leaf was allowed to assimilate ^13^C under natural light conditions for 30 min between 09:30–10:30. Parts of plants were harvested and separated into seven components, the second leaf blade (L2), the second leaf sheath (LS), uppermost internode (INT1), the second internode (INT2), the third internode (INT3), panicle (P), and other organs. The roots were omitted because their quantity was negligible [31, 52]. All divided organs were either freeze-dried or oven-dried, and then ground into a fine powder. Approximately 1.0 mg of well-mixed powder of each tissue was used for the determination of total carbon and the isotopic ratio of ^12^C to ^13^C using an element analyser/isotope ratio mass spectrometer (ANCA-SL, SerCon Company, Cheshire, UK). Whole-plant ^13^C quantity was estimated according to a previous study [52]. Prefixed INT3 were harvested at 6 DAH and 16 DAH. The extraction of the starch fraction prior to ^13^C isotope analysis was conducted according to the previous research [31]. Simply, the sample powder was homogenized with water/ethanol (1:4, v/v). After centrifuging (20 min at 3000 × *g*), starch in insoluble residue was washed with distilled water, and thereafter solubilized starch was hydrolysed to Glc by adding 100 αL α-amyloglucosidase (EC 3.2.1.3) from *Rhizopus* mould (16.7 αg L^-1^, A-7255, Sigma-Aldrich) dissolved in 50 mM sodium acetate buffer (pH 4.5) to 4 mL of starch solution in a water bath at 55°C for 1.42 min, based on the fact that enzyme activity exhibited no varietal dependence at least to 4 min (Fig 4D). α-amyloglucosidase from *Rhizopus* mould was capable of hydrolysing the α-D-(1–4), α-D-(1–6), and α-D-(1–3) glucosidic bonds of oligosaccharides to convert starch to Glc. The reaction was stopped by cooling on ice, and the supernatant was decanted, collected as a fraction, and subsequently the same reaction was repeated for up to 31 fractions. Sodiated Glc, which yielded the most abundant ionic species, including Glc, was analysed using an Orbitrap mass spectrometer (Exactive Orbitrap LC-MS, Thermo Fisher Scientific, Inc.). The ^13^C/^12^C isotope ratio (*m/z* = 204 [C_5_^13^C H_12_ O_6_ + ^23^Na]; Mm. 204.0561693 and *m/z* = 203 [C_6_ H_12_ O_6_ + ^23^Na]; Mm. 203.0527693) was determined according to ref. [20]. Additionally, starch content in the stems and INT3 was determined with an F-Kit (R-Biopharm, Darmstadt, Germany) according to the manufacturer's instructions.

### Plant water status measurements

INT3 and flag leaf samples in the two field-grown HYVs were randomly collected between 07:00–08:00 at -10, -1, 4, 9, 18, and 32 DAH in 2014. They were immediately placed into aluminized Mylar zip-top bags to prevent water loss and rapidly transferred to a humidity box for further dissection. All subsequent tissue manipulations were performed under saturating humidity inside the box to minimize water loss from the tissue after excision [5]. A part (ca. 1 cm) of the mature region of INT3 (segments positioned at approximately 1–2 cm below the junction of INT2/INT3) and the middle part of flag leaves (approximately 1.0 cm^2^) were then gently excised. The water status of the tissues was determined with the isopiestic psychrometers [5, 31, 53]. After water potential was measured, osmotic potential of the tissue was determined in the same tissue immediately after freezing at -80°C and thawing at 25°C. Turgor pressure was calculated by subtracting osmotic potential from water potential [5, 53].

### Isolation of total RNA and genomic DNA

INT3 was excised from the main stem at 07:00–08:00 on each sampling day, immediately frozen in liquid nitrogen, and then stored at -80°C for the following processing. A tissue segment of 2–4 cm from the top of the third internode was removed and crushed into a fine powder with an SK-Mill (Tokken Inc., Chiba, Japan). Total RNA of the internode segment was extracted using RNAs-ici!-S (Rizo Inc., Tsukuba, Japan). The isolated RNA was treated with TURBO DNase (Life Technologies), then precipitated with ethachinmate (NIPPON GENE, Tokyo, Japan) and diluted in DEPC water. Genomic DNA was prepared from young seedlings of each cultivar using ISOPLANT (NIPPON GENE, Tokyo, Japan). The extracted DNA was incubated with RNase (final concentration of 10 μg ml^-1^) at 37°C for 30 min, then precipitated with ethachinmate and resuspended in water.

### Gene expression analysis

Total RNA of 1.5 μg (mixed each 0.5 μg total RNA from 3 independent plants in the same block) was reverse-transcribed using an iScript Advanced cDNA Synthesis kit (Bio-Rad) in a total volume of 30 μL. Quantitative real-time polymerase chain reaction (PCR) was carried out in a real-time PCR system (CFX 96 Connect, Bio-Rad). Each reaction (5 μL) contained 300 nM of each primer, 0.5 μL of 1:50 diluted cDNA, and 2.5 μL of SsoAdvanced SYBR Green Supermix (Bio-Rad). The thermal cycling conditions were 98°C for 3 min followed by 95°C for 5 s and 60°C for 10 s for 45 cycles. Dissociation curves for each amplicon were then obtained by heating from 65 to 95°C. To produce a standard curve of ubiquitin gene (*UBQ5)* as a reference, the genomic DNA was diluted to obtain a series at 1 log_10_ intervals. Each PCR was run in duplicate or triplicate within the same plate, and the C_T_ values obtained from the technical replicates were averaged. Copies of target genes were determined using genomic DNA-based absolute quantification: absolute expression normalized to the absolute level of reference gene expression [30], then averaged for three blocks. Expression level per cell was calculated with an assumption that 1μg RNA corresponds to 5.0 x 10^4^ cells, according to ref. [21]. Primer sequences used for the amplification are listed in S4 Table. In some major genes, rates of absolute gene expression (*ER*s) were determined by dividing changes in expression (*dk*) by time intervals (*dt*) with the curves fitted with the expression data, and *ER*s were further divided by the expression rate *(k)* collected at each timing to obtain relative expression rates (RERs), i.e., RER = (*dk/dt*)(*1/k*).

### Data analysis

For the yield, each yield component, and within-panicle ripened grains, an analysis of variance (ANOVA) in each year and over all three years was conducted with the cultivar, year, and their interaction as fixed effects and the replications within a year as random effects by using a GLM procedure from JMP (version 12.1.0, SAS Institute Inc., Cary, NC). The year means were compared by using a Tukey’s test. Analysis of all other data was conducted using a Student’s *t*-test in JMP.

## Supporting information

S1 Table Solar radiation and mean temperature at each growth stage between 2012–2014. (PDF)

S2 Table Within-panicle ripened grains of two high-yielding rice cultivars, Hokuriku193 and Momiroman. (PDF)

S3 Table Summary of absolute amounts of all gene expressions analyzed. (PDF)

S4 Table Primer sequences used in quantitative RT-PCR [54]. (PDF)

S1 Fig Time course of changes in panicle dry weight of two high-yielding rice cultivars during development in field-grown plants in 2013. (PDF)

S2 Fig The concentration and amount of NSC in each organ in two high-yielding rice cultivars at 4 DAH. (PDF)

S3 Fig Relative expression rate of two major amylase-related genes, *BAM2* and *AMY2A* plotted against turgor pressure in the mature region of INT3 in two high-yielding rice cultivars. (PDF)

## Abbreviations

AGPase: ADP-glucose pyrophosphorylase
AQP: water-transporting proteins
BE: branching enzyme
DAH: days after heading
ΔP: changes in turgor pressure
ESI: electrospray ionization
GBSS2: granule-bound starch synthase II
GWD: α-glucan, water dikinase
H193: Hokuriku 193
HYV: high-yielding variety
INT3: third internode
MEX: maltose exporter
MOMI: Momiroman
MS: mass spectrometry
NSC: non-structural carbohydrates
QTL: quantitative trait locus
SS2b: starch synthesis IIb
SUT: sucrose transporter
pGlcT: plastidial glucose transporter
PWD: phosphoglucan, water dikinase
