# Supplementary material for: Turgor-responsive starch phosphorylation in Oryza sativa stems: A primary event of starch degradation associated with grain-filling ability
Source: PLoS One. 2017 Jul 20;12(7):e0181272. doi: 10.1371/journal.pone.0181272 (PMC5519062; doi:10.1371/journal.pone.0181272)
Supplement: S1 Table — (PDF) [file pone.0181272.s001.pdf]

S1 Table. Solar radiation and mean temperature at each growth stage between 2012-2014. T: transplanting time (mid-June), PI: panicle initiation stage (early August), FH: full heading stage (early September), DAH: days after heading, M: maturity (middle October).

Year	Solar radiation (MJ m^{-2})				Mean temperature (^oC)			
	T-PI	PI-FH	FH-20DAH	21DAH-M	T-PI	PI-FH	FH-20DAH	21DAH-M
2012	16.8	18.3	16.9	15.6	27.1	28.7	26.3	20.2
2013	18.0	21.2	15.6	16.1	27.4	29.7	25.3	24.5
2014	15.0	14.1	16.4	12.8	26.3	26.3	24.9	21.7
