# Supplementary material for: Turgor-responsive starch phosphorylation in Oryza sativa stems: A primary event of starch degradation associated with grain-filling ability
Source: PLoS One. 2017 Jul 20;12(7):e0181272. doi: 10.1371/journal.pone.0181272 (PMC5519062; doi:10.1371/journal.pone.0181272)
Supplement: S2 Table — (PDF) [file pone.0181272.s002.pdf]

S2 Table. Within-panicle ripened grains of two high-yielding rice cultivars, Hokuriku193 and Momiroman. To evaluate the within-panicle filling rate, each panicle was dissected into two portions (upper and lower). Upper and lower in the panicle corresponded to the first to the six, the six to the eleventh primary rachis branches, counting from the uppermost primary rachis branch, respectively. Spikelets in each portion were further classified as classified previously [50]. The tertiary pedicels were isolated from every portion and the rest of the kernels attached to the primary and secondary rachis branches and secondary pedicels were pooled together. Data indicate the mean of three replications. Significant difference at p = 0.01, and 0.001 is indicated by ** and ***, respectively; ns indicates not significant.

		Positions in a panicle			
		Upper position		Lower postion	
Year	Cultivar	Primary and secondary rachis branches and secondary pedicels	Tertiary pedicels	Primary and secondary rachis branches and secondary pedicels	Tertiary pedicels
		%	%	%	%
2012					
	H193	97.3	88.7	92.0	90.5
	Momi	93.8	63.1	90.8	33.5
Cultivar (*p*)			ns		**
2013					
	H193	96.2	91.6	84.9	81.9
	Momi	76.6	68.0	84.0	44.6
Cultivar (*p*)			***		ns
2014					
	H193	92.8	89.5	94.3	84.2
	Momi	92.4	64.3	87.9	24.9
Cultivar (*p*)			ns		**
Cultivar mean					
	H193	95.5	90.0	90.4	85.6
	Momi	87.6	65.1	87.6	34.3
Cultivar (*p*)		ns	***	ns	***
Year effect (*p*)		ns	ns	ns	ns
Cultivar x year (*p*)		ns	ns	ns	ns
