# Supplementary material for: Turgor-responsive starch phosphorylation in Oryza sativa stems: A primary event of starch degradation associated with grain-filling ability
Source: PLoS One. 2017 Jul 20;12(7):e0181272. doi: 10.1371/journal.pone.0181272 (PMC5519062; doi:10.1371/journal.pone.0181272)
Supplement: S3 Table — (PDF) [file pone.0181272.s003.pdf]

S3 Table. Summary of absolute amounts of all gene expressions analyzed in two high-yielding rice cultivars, Hokuriku193 and Momiroman. Mean±SEs (n=3). Significant difference at p = 0.05 and p = 0.01 are indicated by * and **, respectively (t-test).

Gene	Description	Cultivar	Log$_2$ copies ng total RNA^{-1}							
			-1 DAH		4 DAH		9 DAH		18 DAH	
AGPL1	AGPase large subunit 1	Hokuriku 193	16.18 ± 0.48		16.26 ± 0.45		15.00 ± 0.34		10.20 ± 0.25	
		Momiroman	15.64 ± 0.35		16.89 ± 0.46		14.16 ± 1.39		10.30 ± 0.42	
AGPS1	AGPase small subunit 1	Hokuriku 193	15.35 ± 0.56		15.12 ± 0.53		14.41 ± 0.22		11.32 ± 0.23	
		Momiroman	15.66 ± 0.36		16.20 ± 0.32		14.46 ± 0.79		11.48 ± 0.18	
AMY2A	α–amylase 2A	Hokuriku 193	8.32 ± 0.74		10.20 ± 0.29	*	9.38 ± 0.69		11.25 ± 0.25	
		Momiroman	7.32 ± 0.45		6.72 ± 0.72		7.86 ± 0.77		11.22 ± 0.64	
BAM2	β–amylase 2	Hokuriku 193	9.52 ± 0.22		11.98 ± 0.30	*	11.48 ± 0.40		13.88 ± 0.14	**
		Momiroman	8.50 ± 0.32		8.47 ± 0.31		9.66 ± 0.57		12.00 ± 0.36	
BAM3	β–amylase 3	Hokuriku 193	7.46 ± 0.36		7.48 ± 1.14		6.50 ± 0.07		8.33 ± 0.04	
		Momiroman	5.93 ± 1.16		7.36 ± 0.78		5.79 ± 1.25		6.54 ± 0.54	
BAM8	β–amylase 8	Hokuriku 193	8.40 ± 0.56		8.17 ± 0.29		7.63 ± 0.32		7.01 ± 0.12	
		Momiroman	8.31 ± 0.60		8.77 ± 0.09		7.46 ± 0.32		7.00 ± 0.31	
BAM9	β–amylase 9	Hokuriku 193	10.23 ± 0.48		11.90 ± 0.41		11.32 ± 0.10		13.82 ± 0.08	
		Momiroman	10.28 ± 0.24		9.81 ± 0.61		10.83 ± 0.64		13.28 ± 0.23	
BE1	Branching enzyme I	Hokuriku 193	11.29 ± 0.36	*	11.01 ± 0.42	**	10.44 ± 0.21		9.46 ± 0.15	*
		Momiroman	12.55 ± 0.13		13.43 ± 0.29		12.05 ± 0.65		10.15 ± 0.18	
GBSS2	Granule-bound starch synthase II	Hokuriku 193	14.35 ± 0.33		14.21 ± 0.39		13.39 ± 0.16		10.99 ± 0.08	
		Momiroman	14.69 ± 0.12		15.07 ± 0.26		13.37 ± 0.82		10.77 ± 0.32	
GWD1	Glucan, water dikinase 1	Hokuriku 193	12.46 ± 0.57		13.59 ± 0.44	*	12.50 ± 0.20		11.16 ± 0.27	
		Momiroman	10.88 ± 0.26		11.81 ± 0.18		11.11 ± 0.54		10.27 ± 0.17	
ISA3	Isoamylase 3	Hokuriku 193	8.68 ± 0.73		9.43 ± 0.75		8.44 ± 0.10		7.47 ± 0.29	
		Momiroman	8.90 ± 0.47		10.39 ± 0.22		8.69 ± 0.59		8.04 ± 0.05	
MEX1	Chroloplast maltose exporter 1	Hokuriku 193	8.81 ± 0.37		8.75 ± 0.54		7.87 ± 0.21		8.40 ± 0.15	
		Momiroman	9.19 ± 0.25		9.56 ± 0.16		8.34 ± 0.62		8.02 ± 0.11	
pGlcT	Plastidial glucose transporter	Hokuriku 193	8.52 ± 0.56		8.97 ± 0.46		8.67 ± 0.03		8.78 ± 0.10	
		Momiroman	9.15 ± 0.19		9.66 ± 0.21		8.39 ± 0.30		8.89 ± 0.25	
PWD-like	Phosphoglucan, water dikinase-like protein	Hokuriku 193	7.70 ± 0.92		8.67 ± 0.40		8.16 ± 0.14		8.75 ± 0.17	
		Momiroman	7.91 ± 0.25		8.31 ± 0.36		8.07 ± 0.16		8.40 ± 0.16	
SS2b	Starch synthase IIb	Hokuriku 193	11.45 ± 0.55		11.60 ± 0.51		10.89 ± 0.01		10.51 ± 0.31	
		Momiroman	12.46 ± 0.27		12.73 ± 0.27		11.05 ± 0.58		10.22 ± 0.07	
SUT1	Sucrose transporter 1	Hokuriku 193	8.17 ± 0.46		8.80 ± 0.45		8.27 ± 0.18		10.49 ± 0.25	
		Momiroman	9.52 ± 0.33		9.69 ± 0.69		9.15 ± 0.24		10.61 ± 0.24	
