# Supplementary material for: Turgor-responsive starch phosphorylation in Oryza sativa stems: A primary event of starch degradation associated with grain-filling ability
Source: PLoS One. 2017 Jul 20;12(7):e0181272. doi: 10.1371/journal.pone.0181272 (PMC5519062; doi:10.1371/journal.pone.0181272)
Supplement: S4 Table — (PDF) [file pone.0181272.s004.pdf]

S4 Table. Primer sequences used in quantitative RT-PCR. *Ref.[54].

Gene	Locus ID	Forward primer (5'-3')	Reverse primer (5'-3')
AGPL1	Os05g0580000	GTCATCACAAACAGCGAGGGTG	GAGATCTGTGGCATCAGCGAG
AGPS1	Os09g0298200	ACGCCTTAATCCCTAGCGGAAC	TGCTGCAAGGCCCAACCTTATG
AMY2A	Os06g0713800	CGTCGCACTCCGTCTCCAC	GATCAACGACTTCAACTCCATC
BAM2	Os10g0465700	ATGGAGATGGCCCGCAAGAC	ACCTCGGGAGCGGGATGTTG
BAM3	Os03g0141200	TCGGCGACTCAGTCACTATACCAC	AGCGAGAGGTATTCGTAGTTGCG
BAM8	Os09g0569200	CGGTTACAAGCGACTCTTCC	CTTCCGATCTCTGTTACCCAATG
BAM9	Os03g0351300	CCGCCTTCCATGACTTCTTG	CGGATACGAAGGGTACTTGAG
BE1	Os06g0726400	TTGGTGGCCATGGAAGAGTTGG	TTCTGGTACTCCTGGCATTCCC
GBSS2	Os07g0412100	AGGGATGAGATATGGAGTGGTTC	CGTCCACTGGATCAACAGTTTC
GWD1	Os06g0498400	GCGAAATACGGAAAACTGTTCTC	GTTGGTCACCTTCATCTCCAG
ISA3	Os09g0469400	CAATGCACATGACTATTTTGTGGAC	AGCAGAATGGAAGAGTATGGAGC
MEX1	Os04g0602400	TTTCTTGGCAACAACATTTGGAC	CTTCAAAGAAGTCACGGGTGAG
*pGlcT**	Os01g0133400	TGGAGCTTCGAGGGTTGTGA	CGGTGGCATACTGGCATCTA
PWD-like	Os12g0297500	CTCTTTCTGTTCTTGCTTCATTGTC	CTTGAGCGCATCCTCCATTG
SS2b	Os02g0744700	ACAACATTGCTCATCAGGGTCGTG	ATCGATGTAGTGCTCGGGCAAG
SUT1	Os03g0170900	TGCCTCGTCCTCTTTGCGTTTC	GCGATGACCACCTGAGGGATG
UBQ5	Os01g0328400	CAGGCTTAGGCGTAGGCTC	CATCTAATAACCAGTTCGATTTCC
