# Supplementary material for: Turgor-responsive starch phosphorylation in Oryza sativa stems: A primary event of starch degradation associated with grain-filling ability
Source: PLoS One. 2017 Jul 20;12(7):e0181272. doi: 10.1371/journal.pone.0181272 (PMC5519062; doi:10.1371/journal.pone.0181272)
Supplement: S1 Fig — (PDF) [file pone.0181272.s005.pdf]

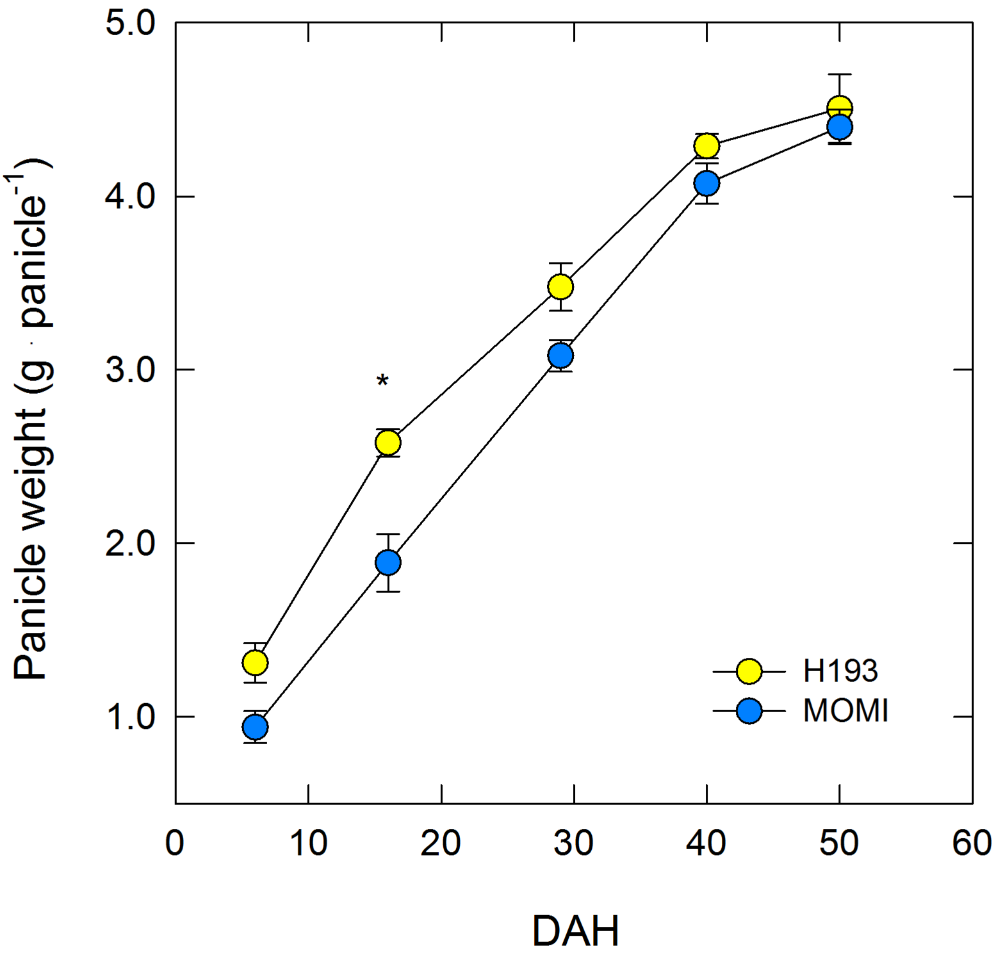

S1 Figure. **Time course of changes in panicle dry weight of two high-yielding rice cultivars during development in field-grown plants in 2013.** Yellow and light blue circles indicate H193 and MOMI, respectively. Data are the mean±SEs (n=3) from the panicles collected from three independently repeated experimental plots. Significance at the 0.05 probability level is indicated with *.
