# Supplementary material for: Turgor-responsive starch phosphorylation in Oryza sativa stems: A primary event of starch degradation associated with grain-filling ability
Source: PLoS One. 2017 Jul 20;12(7):e0181272. doi: 10.1371/journal.pone.0181272 (PMC5519062; doi:10.1371/journal.pone.0181272)
Supplement: S2 Fig — (PDF) [file pone.0181272.s006.pdf]

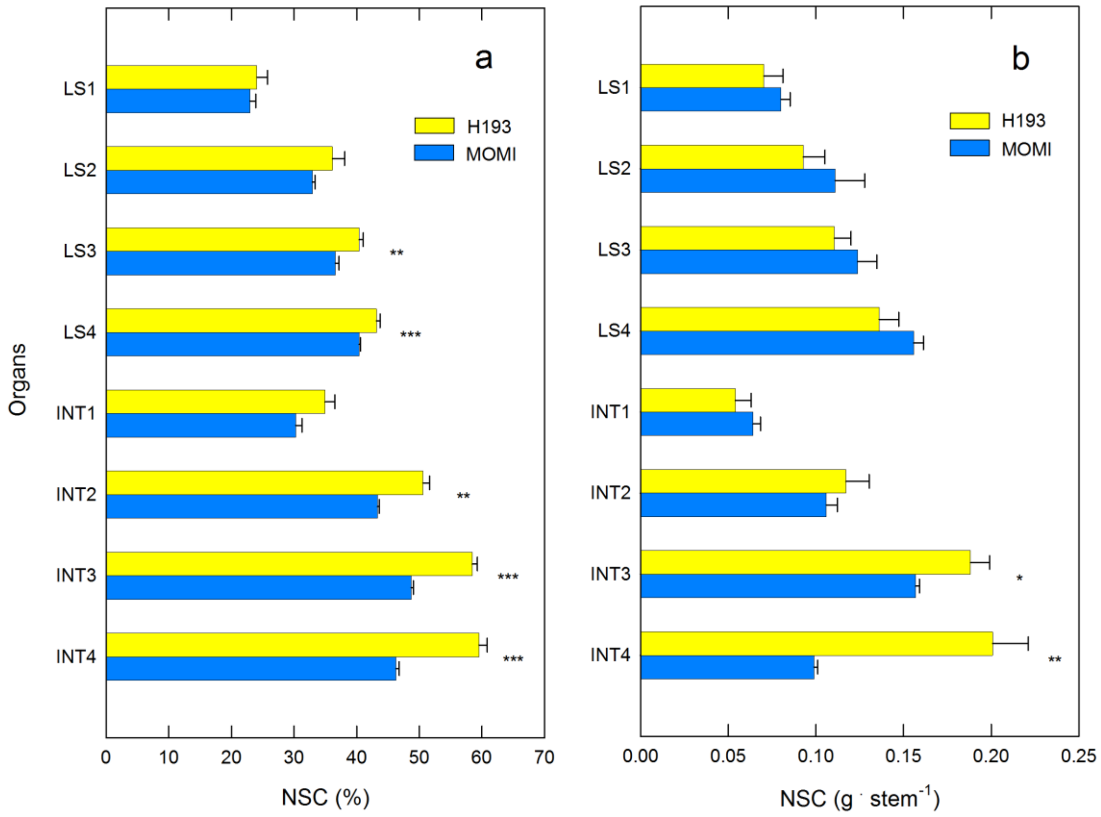

S2 Figure. **The concentration and amount of nonstructural carbohydrate (NSC) in each organ in two high-yielding rice cultivars at 4 DAH.** a and b indicate the concentration and amount of NSC, respectively. Yellow and light blue bars indicate H193 and MOMI, respectively. The data show that INT3 was one of the highest NSC storage organs in both cultivars, corresponding to 18-19% of the entire stem NSC. Data are the mean±SEs (n=3) from the pooled 3 field-grown plants collected from three independently repeated experimental plots. Significance at the 0.05, 0.01 and 0.001 probability levels is indicated with *, **, and ***, respectively.
