# Supplementary material for: Turgor-responsive starch phosphorylation in Oryza sativa stems: A primary event of starch degradation associated with grain-filling ability
Source: PLoS One. 2017 Jul 20;12(7):e0181272. doi: 10.1371/journal.pone.0181272 (PMC5519062; doi:10.1371/journal.pone.0181272)
Supplement: S3 Fig — (PDF) [file pone.0181272.s007.pdf]

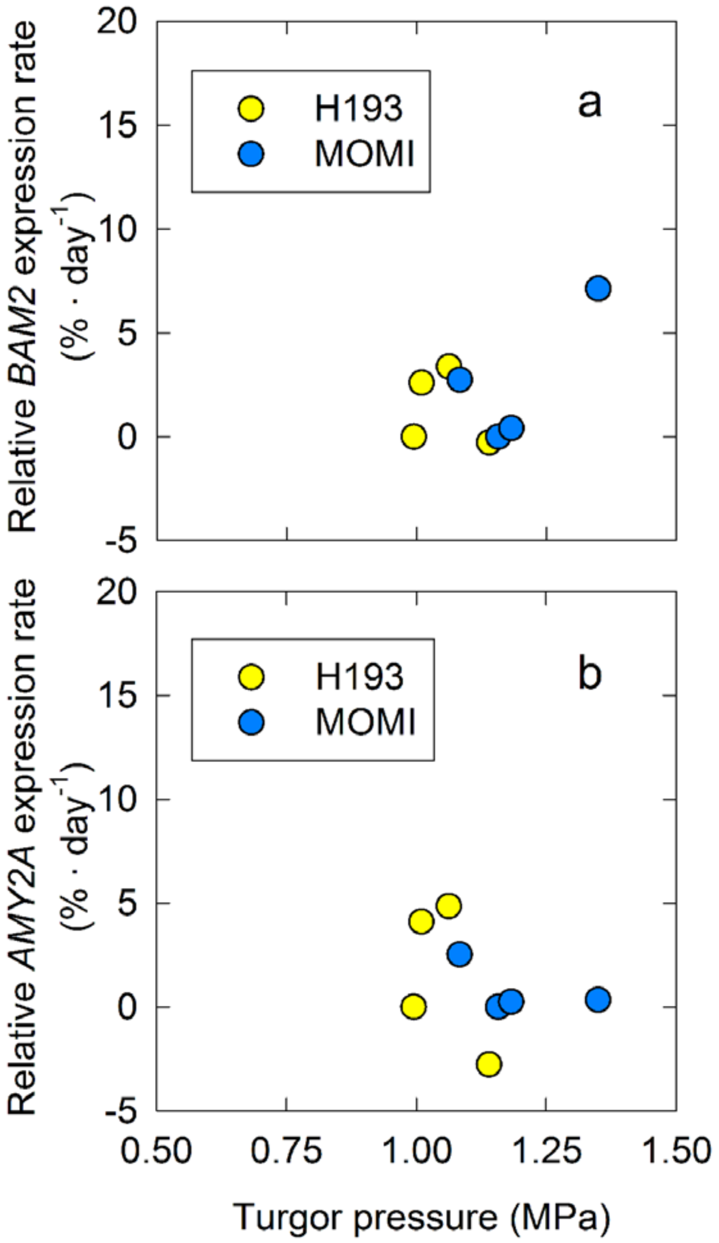

S3 Figure. **Relative expression rate of two major amylase-related genes, *BAM2* (a) and *AMY2A* (b) plotted against turgor pressure in the mature region of INT3 in two high-yielding rice cultivars.** Yellow and light blue symbols indicate H193 and MOMI, respectively.
